# Icariside II enhances cisplatin-induced apoptosis by promoting endoplasmic reticulum stress signalling in non-small cell lung cancer cells

**DOI:** 10.7150/ijbs.66630

**Published:** 2022-02-28

**Authors:** Zhao Tang, Wenjing Du, Fei Xu, Xianjun Sun, Wenjing Chen, Jie Cui, Weifeng Tang, Fangyong Yang, Fangzhou Teng, Jinpei Lin, Baojun Liu, Jingcheng Dong

**Affiliations:** 1 Department of Integrative Medicine, Huashan Hospital, Fudan University, Shanghai, China.; 2 Institutes of Integrative Medicine, Fudan University, Shanghai, China.; 3 Department of Integrated TCM & Western Medicine Department, Fujian Cancer Hospital & Fujian Medical University Cancer Hospital, Fuzhou, China.

**Keywords:** icariside II, lung cancer, cisplatin, apoptosis, endoplasmic reticulum stress, unfolded protein response

## Abstract

Although cisplatin is the most effective first-line drug in the management of advanced non-small cell lung cancer (NSCLC), drug resistance remains a major clinical challenge. There is increasing evidence that icariside II (IS) exhibits antitumour activity in a variety of cancers. In the current study, we investigated the anticancer effects of icariside II combined with cisplatin and elucidated the underlying mechanism in NSCLC. Here, we showed that cotreatment with IS and cisplatin inhibited cell proliferation and induced cellular apoptosis. Using mRNA sequencing (mRNA-seq), we identified differentially expressed genes (DEGs) in which there was an enrichment in PERK-mediated unfolded protein response (UPR) signalling. The western blot results revealed that IS activated endoplasmic reticulum (ER) stress, including three branches of UPR signalling, PERK, IRE1 and ATF6, and the downstream PERK-eIF2α-ATF4-CHOP pathway, thus potentiating the apoptosis induced by cisplatin. In addition, the combination of IS with cisplatin significantly reduced xenograft tumour growth in C57BL/6 and BALB/c nude mice *in vivo*. Notably, the combination therapy displayed no evident toxicity. Taken together, IS enhances cisplatin-induced apoptosis partially by promoting ER stress signalling in NSCLC, suggesting that combination treatment with IS and cisplatin is a novel potential therapeutic strategy for NSCLC.

## Introduction

Lung cancer is one of the leading causes of cancer mortality around the world, with an estimated 11.4% of new cases and 18% of deaths in 2020 1. The approximately 85% of lung cancers are non-small cell lung cancer (NSCLC), with dismal median overall survival and 5-year survival rates due to recurrence, extensive invasion and metastasis 2-4. Cisplatin (DDP) is a first-line cytotoxic agent for the treatment of human NSCLC. However, patients receiving cisplatin-based chemotherapy often develop resistance, which diminishes its efficacy and limits the clinical utility of this drug 5. Therefore, the development of effective strategies to reduce cisplatin chemoresistance is highly desired in the clinic.

Icariside II (IS) is an active flavonoid glycoside derived from the traditional Chinese drug *Herba Epimedii*. Previous studies suggested that it possesses multiple physiological and pharmaceutical functions, including anti-inflammatory, antioxidant, immunomodulatory, neuroprotective and cardioprotective effects 6-11. In addition, IS has been demonstrated to exhibit antitumour activities against various cancers, such as cervical cancer, hepatocellular carcinoma and melanoma, through different signalling pathways 12-15. Nevertheless, most previous studies focused only on a single use of IS, and little is known about combination therapy of IS and cisplatin in NSCLC.

Cisplatin binds to DNA, therefore producing inter- or intrastrand cross-links between adjacent purines, which inhibit DNA replication and transcription, consequently leading to apoptosis 16. During apoptosis, activation of the caspase cascade is a key event, and caspase-3 is the main effector caspase involved in cisplatin-induced apoptosis. The Bcl-2 family contains essential apoptosis regulators, including Bcl-2 and Bax 17.

Endoplasmic reticulum (ER) stress generally occurs in tumours due to stressful extracellular environmental challenges, such as hypoxia, nutrient deprivation, and pH changes. Moderate ER stress contributes to cancer cell survival and chemotherapeutic resistance; however, excessive and prolonged ER stress results in apoptosis 18-19. Cancer cells stimulate the unfolded protein response (UPR) as an adaptive program to maintain ER homeostasis. The UPR is a general term for multiple signal transduction pathways, including three ER transmembrane proteins: ATF6, PERK and IRE1. Following ER stress, PERK and IRE1α (referred to as IRE1) are activated by phosphorylation, while ATF6 is delivered from the ER to the Golgi apparatus where it is cut to remove an N-terminal fragment and then it releases its cytosolic domain 20. UPR induction is a natural outcome of ER stress. The initial UPR can promote the clearance of unfolded and misfolded proteins and is an adaptive program to re-establish ER homeostasis to maintain cellular viability. However, when the UPR fails to relieve ER stress, many proapoptotic signalling pathways, such as CHOP upregulation, JNK activation and caspase-12 cleavage, are initiated 21-22.

In this study, we evaluated whether IS could potentiate the antitumour activity of cisplatin in mice and preliminarily examined the safety of the combined therapy. Moreover, the effect of the combination of IS with cisplatin on apoptosis and ER stress was analysed *in vitro* using four lung cancer cell lines. Our findings revealed a novel molecular mechanism underlying the anticancer effects of a combination of IS and cisplatin, which may provide a potential treatment strategy for lung cancer.

## Materials and methods

### Reagents and cell lines

Shanghai Winherb Medical Technology Co., Ltd. provided icariside II (purity > 98%). Cisplatin was purchased from Sigma-Aldrich, which was diluted in sterile 0.9% sodium chloride. The apoptosis kit was obtained from BD Bioscience (CA, USA). 4-phenylbutyric acid (4-PBA) was obtained from Sigma-Aldrich. Lewis lung carcinoma (LLC) cells, NSCLC cells A549 and H1299 and cisplatin-resistant A549 (A549/DDP) cells were obtained from the Shanghai Chinese Academy of Sciences cell bank.

### Animal model and treatment

Male BALB/c and C57BL/6 nude mice (aged 4-5 weeks) were obtained from Shanghai SIPPR-BK Laboratory Animal Co., Ltd. All animal experiments were performed with the approval of the Experimental Animal Ethics Committee of School of Pharmacy, Fudan University. C57BL/6 mice received a subcutaneous injection of 4×10^5^ LLC cells into the right flank, while BALB/c mice received 6×10^6^ A549 cells. On Days 7 and 11, tissue masses were palpable at the site of injection in C57BL/6 and BALB/c mice, respectively. To examine the effect of IS and cisplatin on tumour growth, the mice were divided into four groups at random (n = 5): control group, IS, cisplatin, IS and cisplatin. Cisplatin (3 mg/kg) was administered intraperitoneally every 3 days, while IS (40 mg/kg) suspended via sonication in 0.5% CMC-Na was administered by oral gavage daily. The mice were sacrificed at 2 weeks, and their blood, primary tumour, heart, liver, and kidney tissues were harvested. The measurement of the body weights and tumour volumes were performed every 3 days. The tumour growth inhibition rate and the interactions between IS and cisplatin was done as previously reported 23, 24.

Inhibition rate (%) = (1 - mean tumor weight of the treatment group/mean tumor weight of the control group) × 100%. The interactions between IS and cisplatin were measured using *q* value as the formula: *q* = E_A+B_ / (E_A_ + E_B_ - E_A_ × E_B_), where E_A+B_, E_A_ and E_B_ represent the inhibition effect of combination treatment, IS and cisplatin, respectively. The interactions include antagonistic effect (*q* < 0.85), additive effect (0.85 < *q* < 1.15) or synergistic effect (*q* > 1.15).

### Cytotoxicity assays

The cytotoxicity of IS and cisplatin was determined by MTT assays. LLC, H1299, A549 and A549/DDP cells were co-treated with IS or cisplatin as indicated at different doses. After 24-72 h, 10 μl MTT reagent (Sigma-Aldrich) was added to each well, the mixture was incubated for 4 h.The absorbance at 490 nm was recorded and plotted using an MK3 ELISA reader.

### Flow cytometry

Cancer cells incubated with IS, cisplatin or IS plus cisplatin for 48 h were collected and detected using an Annexin V-FITC/PI Kit (BD Biosciences, USA). The percentage of apoptotic cells was analyzed based on the population of early and late apoptotic cells.

### mRNA sequencing (mRNA-seq)

Total RNA from A549/DDP cells treated with vehicle, IS (40 μM) and/or cisplatin (150 μM) was extracted using an RNA isolation kit (Invitrogen). The RNA quality was analysed using capillary electrophoresis with an Agilent 4200 Tapestation system prior to library preparation. The adaptor-ligated libraries were paired-end sequenced on the Illumina NovaSeq 6000 system (Illumina). The reads were mapped to the reference human genome with HISAT26. GO and KEGG pathway enrichment analyses of differentially expressed genes (DEGs) were implemented with the R package.

### Transmission electron microscopy (TEM)

Tumour cells were fixed and postfixed in 2% glutaraldehyde overnight and 2% osmium tetroxide for 90 min, respectively. After dehydration and embedding, sample sections were cut and stained with lead citrate. Ultrathin sections were stained with uranyl acetate and lead citrate. Imaging of the slices was acquired with a Libra 120 transmission electron microscope (Zeiss).

### Western blot

Cellular proteins were extracted from tumour tissues and lung cancer cells using RIPA lysis buffer. The proteins was separated on a 5-15% SDS-PAGE gel,then transferred to PVDF membranes, blocked with 5% non-fat milk and incubated with the indicated primary antibodies, including antibodies against cleaved caspase-3, PERK, ATF6, eIF2α, phospho-eIF2α, CHOP, phospho-JNK and β-actin (CST, USA) and antibodies against phospho-PERK and ATF4 (Affinity Biosciences) and phospho-IRE1α (Abcam, USA), overnight at 4 °C. Subsequently, the membranes were incubated with secondary antibodies at room temperature for 1.5 h. The immune signals were visualized with an ECL kit.

### Statistical analysis

The values are given as the mean ± SD. Statistical analyses were conducted with one-way ANOVA, followed by Tukey's post-hoc test or Student's t-test using GraphPad Prism 8 software. Statistical significance was considered *P* < 0.05.

## Results

### Combined treatment of IS and cisplatin inhibits the proliferation of lung cancer cells

**Figure 1:**
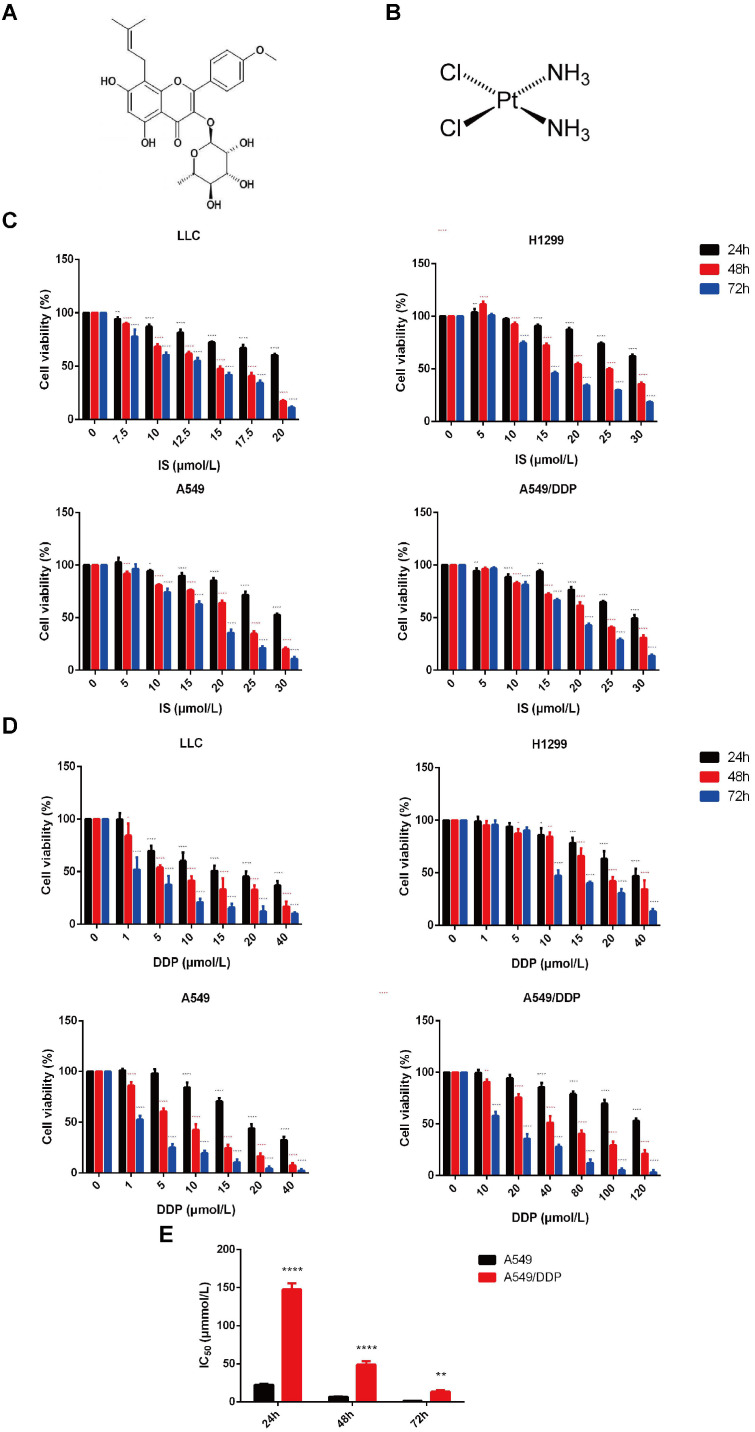
** The cytotoxic effect of IS and cisplatin on LLC, H1299, A549 and A549/DDP cells.** The chemical structural formulas of IS **(A)** and cisplatin **(B)** are shown. **(C)** The cells were cultured with various concentrations of IS for 24, 48 or 72 h, and cell viability was detected by MTT assays. **(D)** The viability of cells treated with cisplatin for 24, 48 or 72 h. **(E)** The IC_50_ values for cisplatin in A549/DDP cells compared with A549 cells. The resistance index (RI) was calculated using the formula: IR = IC_50_ (resistant cells)/IC_50_ (sensitive cells). IS: icariside II; DDP = cisplatin. Data are shown as the mean ± SD, n = 3, *P < 0.05, **P < 0.01, ***P < 0.001, ****P < 0.0001.

Four lung cancer cell lines, LLC, H1299, and A549, and cisplatin-resistant A549/DDP cells, were adopted for the *in vitro* experiments. The sensitivity of these cells to IS and cisplatin was examined by MTT assay. The cytotoxic effects of both IS and cisplatin were enhanced gradually in a dose- and time-dependent manner (Figure 1A-D). The viability of the cells gradually decreased with increasing treatment time and concentration, and the inhibition levels of IS and cisplatin differed in different cells (Table 1). The A549/DDP cells displayed resistance to cisplatin treatment, with drug resistance index values of 6.61 (24 h), 7.27 (48 h) and 11.16 (72 h) (Figure 1E).

**Figure 2:**
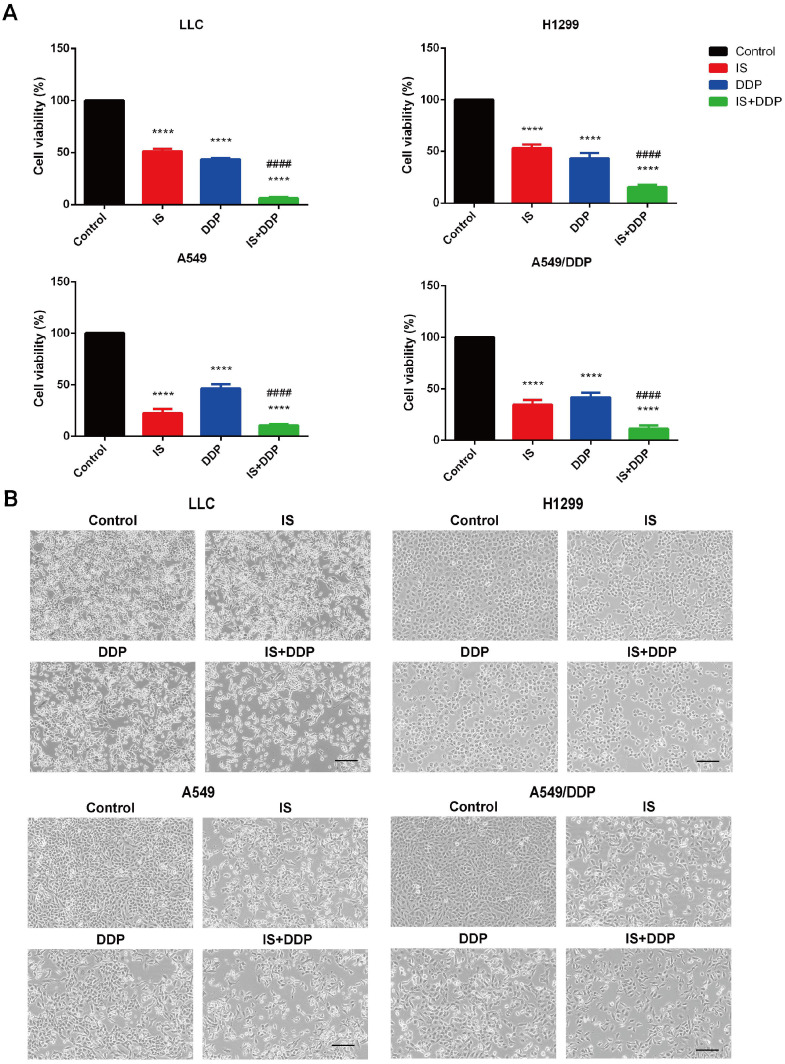
** IS combined with cisplatin inhibits NSCLC cell proliferation* in vitro*.** Cells were treated with IS in combination with cisplatin for 48 h (LLC: IS 15 µmol/L + DDP 10 µmol/; H1299: IS 25 µmol/L + DDP 25 µmol/L; A549: IS 30 µmol/L + DDP 10 µmol/L; A549/DDP: IS 30 µmol/L + DDP 50 µmol/L). The data were from at least three independent experiments. **(A)** Percentage inhibition of each cell line. ****P < 0.0001 compared with the control, ####*P* < 0.0001 compared with the cisplatin group. **(B)** Representative brightfield images. Scale bar = 100 µm.

Then, we treated the cells with IS with or without cisplatin. The treatment concentration was determined based on the half maximal inhibitory concentration (IC_50_) values calculated from the MTT results. Compared with cisplatin treatment alone, combined treatment obviously slowed cell proliferation, with decreased cell attachment at lower densities and many more floating cells in the culture medium (Figure 2A-B). IS increased the antiproliferative effect of cisplatin against these lung cancer cell lines.

### IS in combination with cisplatin induces apoptosis

**Figure 3:**
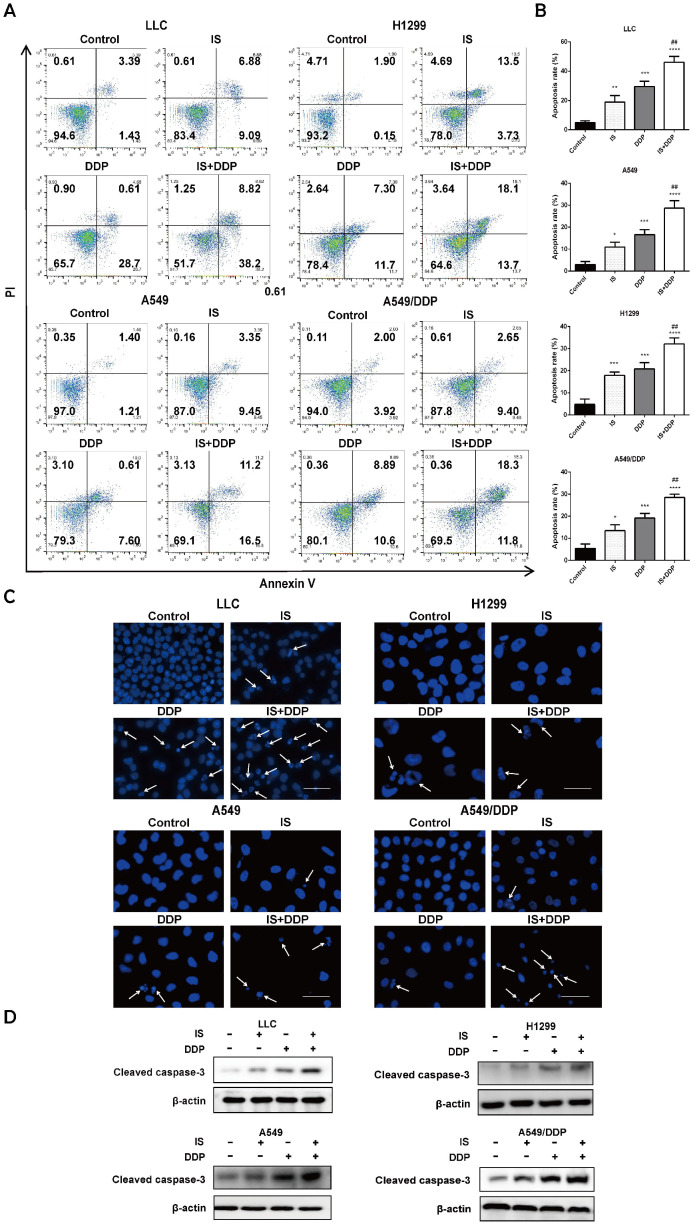
** IS in combination with cisplatin induces apoptosis of cells.** Cells were treated with IS with or without cisplatin at the indicated concentrations for 48 h. IS promotes apoptosis, as shown by flow cytometry (**A-B**) and DAPI staining (**C**). **(D)** IS combined with cisplatin increased the levels of the cleaved fragments of caspase-3 compared with cisplatin treatment alone. Cells were treated with IS in combination with cisplatin for 24 h (LLC: IS 25 µmol/L + DDP 20 µmol/; H1299: IS 35 µmol/L + DDP 40 µmol/L; A549: IS 40 µmol/L + DDP 30 µmol/L; A549/DDP: IS 40 µmol/L + DDP 150 µmol/L). The experiments were performed at least three times. *P < 0.05, ***P < 0.001, ****P < 0.0001 compared with the control, ##*P* < 0.01 compared with the cisplatin group.

As shown by flow cytometric examination of different cell populations treated with IS, cisplatin and IS + cisplatin, the proportion of apoptotic cells in the combination groups was the highest (Figure 3A-B). Compared to the uniformly stained blue fluorescence of nuclei in the control group, the cells exhibited nuclear pyknosis, condensation, and fragmentation after exposure to cisplatin for 48 h (Figure 3C). The changes resulting from cisplatin were enhanced by IS, and this was further supported by the increased cleavage of caspase-3 evident on western blotting (Figure 3D). IS enhanced cisplatin-induced apoptosis in LLC, H1299, A549 and A549/DDP cells.

### Global transcriptomic response and IS-mediated signatures in NSCLC

**Figure 4:**
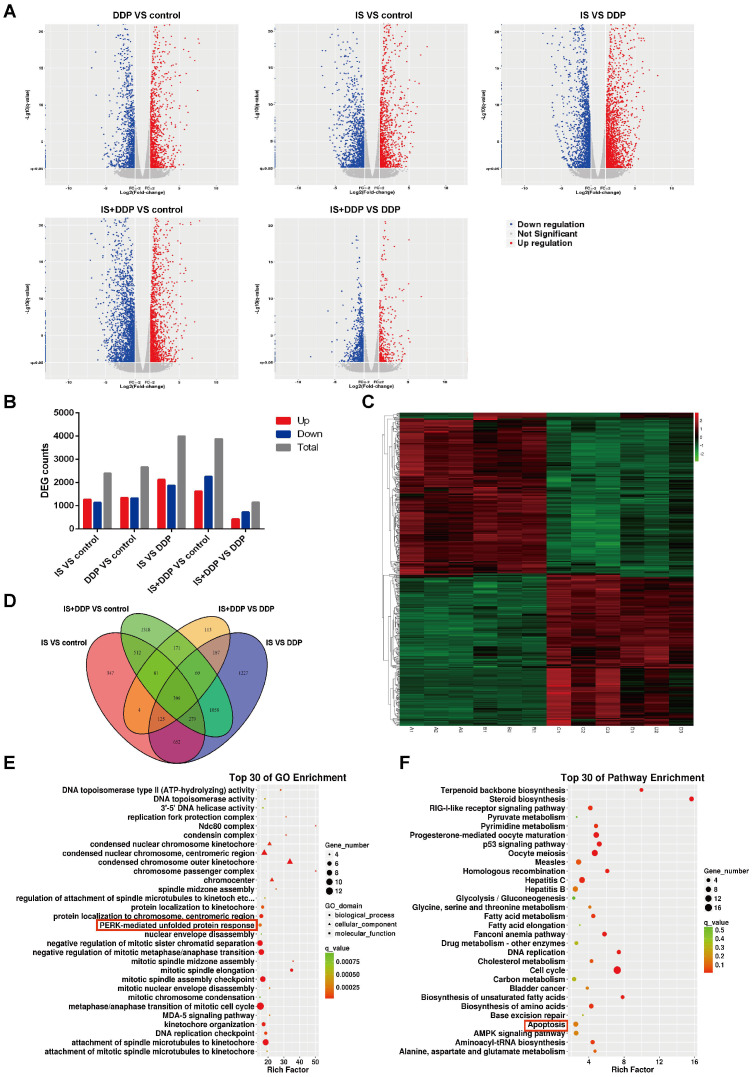
** Transcriptomic profiling of A549/DDP cells treated with IS and cisplatin identified differentially expressed genes (DEGs). (A)** Volcano plot of mRNA expression in DDP vs. control, IS vs. control, IS vs. DDP and IS+DDP vs. DDP. **(B)** DEG counts identified by mRNA-seq. **(C)** Heatmap illustrating the DEGs in the four groups for hierarchical clustering. A = control, B = IS group, C = cisplatin group, D = combination treatment group. **(D)** Venn diagram of overlapping genes identified in the differential expression analysis of the four groups. **(E)** The top 30 enriched GO terms for differentially expressed mRNAs. **(F)** The top 30 significantly enriched KEGG pathways.

To provide an in-depth understanding of the mechanism by which IS combined with cisplatin inhibits NSCLC cell growth, mRNA-seq was conducted to explore genes involved in the antitumour effects of combined therapy. Global differentially expressed gene (DGE) changes among the four groups are shown as volcano plots (Figure 4A). Compared with the control, 2399 and 2668 genes were dramatically altered in the IS and cisplatin-treated groups, respectively. There were 1149 genes with significantly changed DEGs following combination treatment compared with cisplatin treatment alone (Figure 4B). A Venn diagram was used to show the overlaps of the 399 significantly repressed DEGs among the groups (Figure 4C-D). According to Gene Ontology (GO) and KEGG analysis, these genes with altered expression were enriched for diverse biological processes and pathways, such as PERK-mediated UPR, apoptosis and DNA replication. The top 30 GO terms and KEGG pathways are shown in Figures 4E and F.

### IS enhances cisplatin-induced apoptosis by promoting ER stress

**Figure 5:**
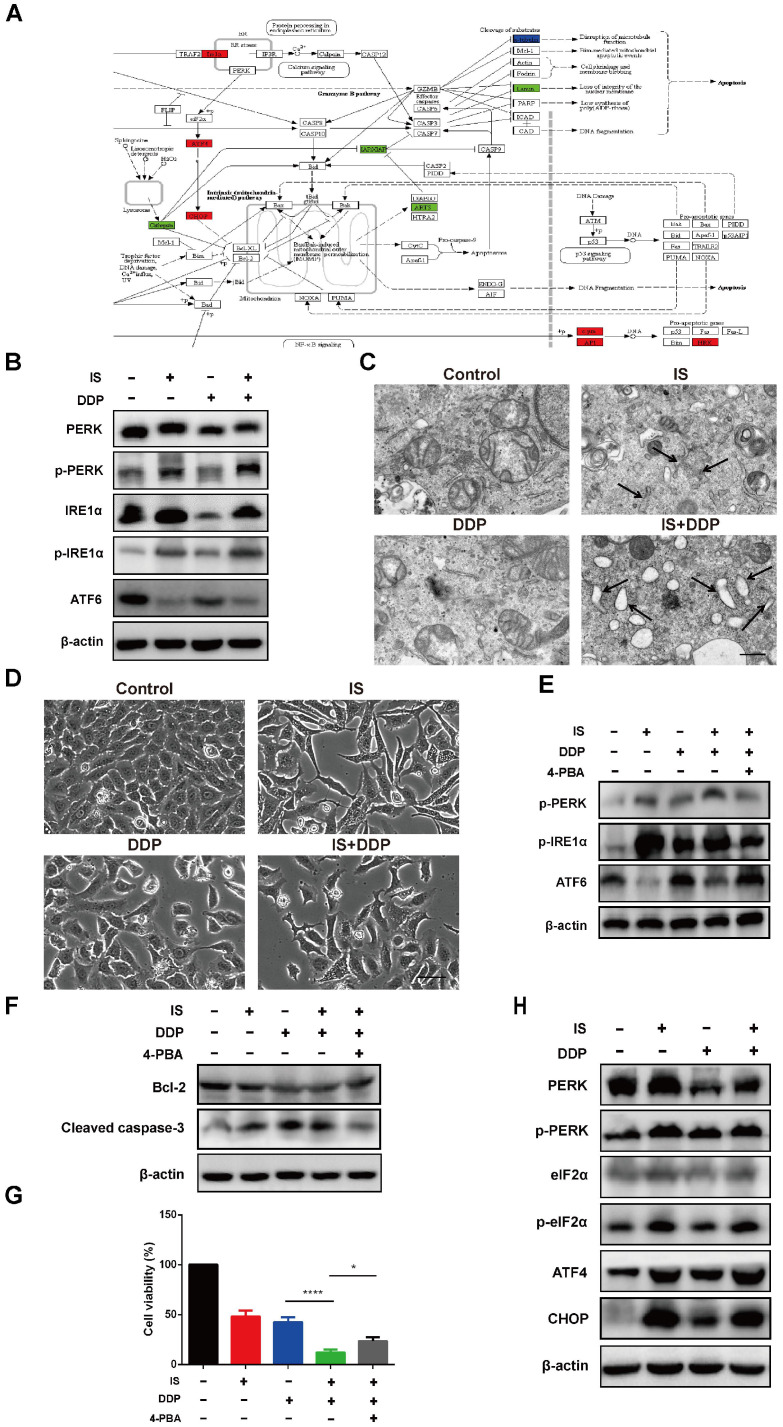
** IS enhances cisplatin-induced apoptosis by promoting ER stress in A549/DDP cells. (A)** KEGG database analyses revealed the effect of IS on the ER stress signalling pathway (coloured box). **(B)** IS activates three UPR signalling branches. A549/DDP cells were treated with IS (40 µmol/L) and/or cisplatin (150 µmol/L) for 24 h. The protein expression levels of PERK, p-PERK, IRE1α, p-IRE1α and ATF6 in A549/DDP cells were measured by western blotting. **(C)** TEM images of A549/DDP cells at 7000× magnification are shown. Black arrows, dilated ER. Scale bar = 5 µm. **(D)** Light microscopy showing that IS induced cytoplasmic vacuolation in A549/DDP cells. Scale bar = 20 µm. **(E)** 4-PBA attenuates the UPR of cells treated with IS and cisplatin. A549/DDP cells were pretreated with 4-PBA (2.5 mmol/L) or PBS (control) for 2 h and then treated with IS, cisplatin or IS + cisplatin for 24 h. **(F)** Inhibition of UPR suppresses apoptosis. **(G)** 4-PBA partially rescued cell viability. *P < 0.05, ****P < 0.0001. (H) IS promotes the expression of the proapoptotic transcription factor CHOP. Cells were harvested after 24 h of treatment. Data were from at least three independent experiments. 4-PBA: 4-phyenylbutyric acid.

GO analysis revealed that IS induced UPR with or without cisplatin. The related DEGs of the UPR pathway were mapped by Pathview (Figure 5A). Activation of the three UPR pathways initiated the adaptive ER stress response. Excessive and sustained ER stress leads to proapoptotic signalling in various human cancers, including NSCLC 25. IS upregulated the expression of PERK, phospho-PERK, IRE1α and phospho-IRE1α while downregulating the level of full-length ATF6. The same tendency in the variation of these factors was observed in the combination treatment group compared with the cisplatin group, indicating that IS plus cisplatin triggered PERK, IRE1α, and ATF6 signalling and promoted ER stress (Figure 5B).

Transmission electron microscopy images demonstrated that untreated A549/DDP cells exhibited a normal appearance with continuous and compact ER with narrow profiles of cisternae. IS resulted in swollen ER cisternae, and cells treated with IS and cisplatin displayed notable ER dilation and disorganization (Figure 5C). The light microscopy findings were in line with these ultrastructural changes, showing that IS combined with cisplatin induced more cytoplasmic vacuolation than cisplatin alone (Figure 5D).

To estimate the role of the UPR in apoptosis induced by IS plus cisplatin, 4-PBA was utilized as a chemical chaperone to prevent activation of the UPR and inhibit ER stress. We found that 4-PBA reversed the expression of phospho-PERK, phospho-IRE1α and ATF6 induced by the combined treatment (Figure 5E). Concurrently, there was also a decrease in the cleavage of caspase-3 and an increase in the expression of Bcl-2, reflecting inhibition of apoptosis, and the cell viability partially recovered (Figure 5F-G). Thus, these results suggested that ER stress might be responsible for IS enhancement of cisplatin-induced apoptosis.

We studied the changes in the PERK-eIF2α-ATF4-CHOP pathway. As shown in Figure 4C, IS and cisplatin combination treatment significantly increased the expression of eIF2α, ATF4, and CHOP and the phosphorylation of eIF2α (Figure 5H). As overexpression of CHOP is associated with stimulation of apoptosis, the PERK-eIF2α-ATF4-CHOP pathway might contribute to apoptosis induced by cotreatment with IS and cisplatin.

### IS combined with cisplatin suppresses lung tumorigenesis *in vivo*

**Figure 6:**
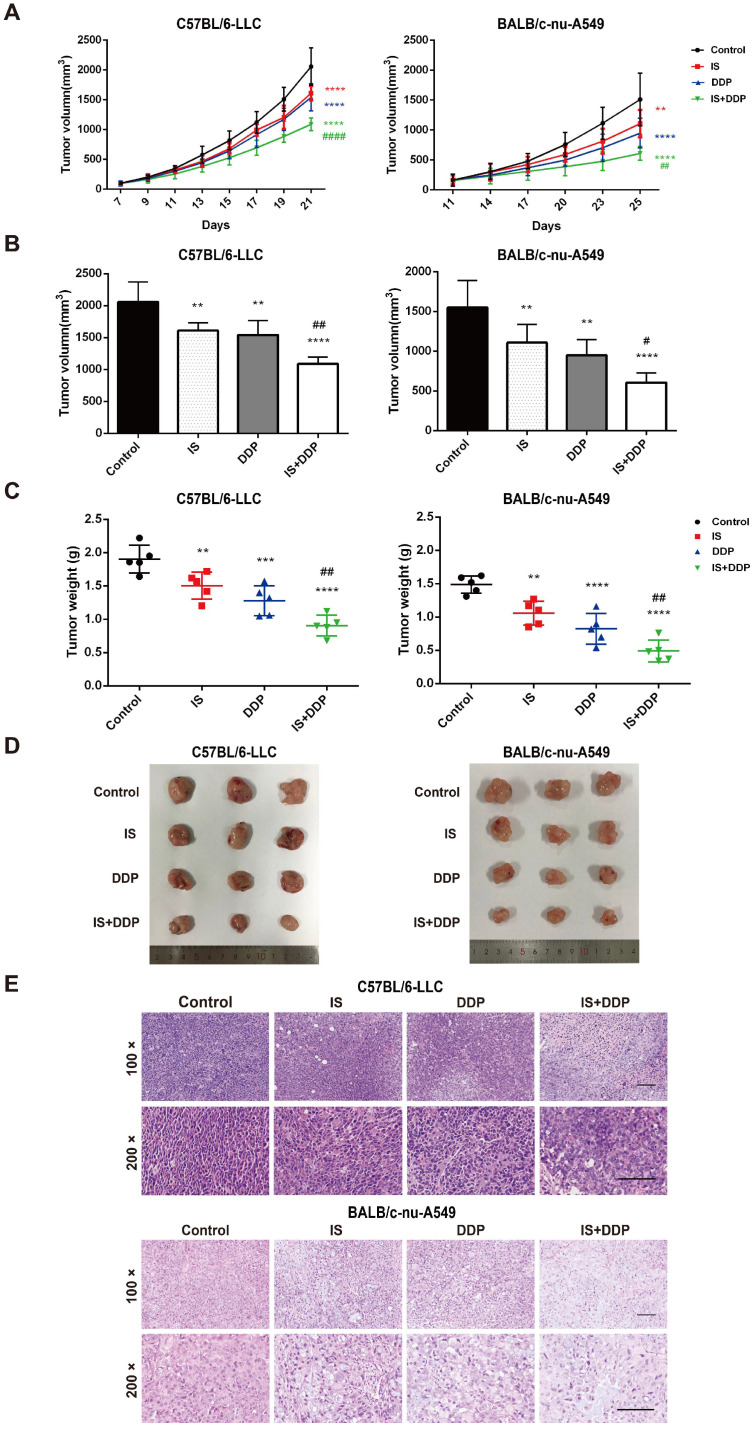
** Combination therapy with IS and cisplatin inhibits lung tumour growth *in vivo*. (A)** The growth curve of the tumour volume (mm^3^) in mice. **(B-C)** The final tumour volume and weight at the time of necropsy. **(D)** Representative images of LLC and A549 xenograft tumours. **(E)** Histological features of tumours examined by HE staining. n = 5 in each group. **P < 0.01, ***P < 0.001, ****P < 0.0001 vs. control group, #*P* < 0.01, ##*P* < 0.01, ####*P* < 0.0001 vs. cisplatin group. Scale bar = 100 µm.

Xenograft tumour models of NSCLC cells were established in C57BL/6 and BALB/c mice to examine the antitumour effect of combined treatment with IS and cisplatin *in vivo*. The mice were treated with IS with or without cisplatin (Figure 6A-D). IS and cisplatin inhibited tumour growth at inhibition rates of 20.53% and 32.63% in C57BL/6 mice and 28.86% and 44.97% in BALB/c mice, respectively, while IS plus cisplatin exhibited significantly enhanced efficacy, with an inhibition rate above 50%. The q values of the combination treatment were 1.12 and 1.1, which indicated an additive effect against the tumours (Table 2).

Tumour tissues in each group were stained with HE and examined by light microscopy. As seen in Figure 6E, tumour cells in the control group tended to be denser and tightly packed and exhibited typical morphological abnormalities, including heterogeneous size and morphology, hyperchromatic nuclei with an increased nuclear size, irregular nuclear membranes, and a high nucleus-to-cytoplasm ratio accompanied by pathological karyokinesis figures. IS or cisplatin treatment showed improvements in cell cluster density, a reduction in heterogeneous size and morphology, and more normal nuclei, nuclear membranes and mitosis. There was partial tumour necrosis in the tissues. The combination treatment significantly decreased the cell density and pathological karyokinesis, increased cell necrosis, and led to a smaller nucleus and shallower colour of staining. Interestingly, more inflammatory cells infiltrated into LLC xenograft tumours in the combination treatment group than in the cisplatin group. Taken together, these observations indicated that IS inhibited tumour growth when given as a single agent and enhanced the chemotherapeutic efficacy of cisplatin *in vivo*.

### Cotreatment with IS and cisplatin induces cell apoptosis and inhibits angiogenesis in xenograft tumours

**Figure 7:**
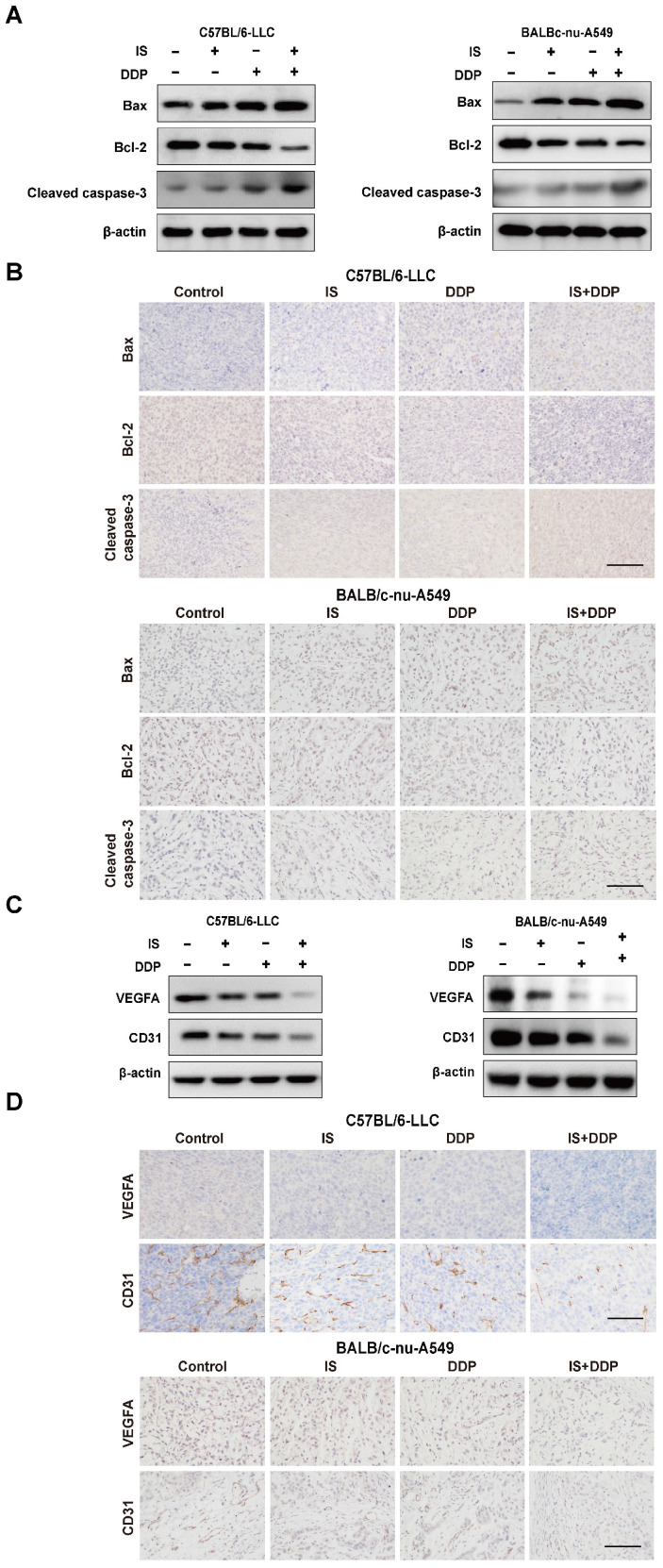
** IS in combination with cisplatin induces apoptosis and inhibits the angiogenesis of tumours. (A-B)** Western blotting and immunohistochemical staining of cleaved caspase-3, Bcl-2 and Bax. **(C-D)** IS enhanced the expression of VEGFA and CD31. Scale bar = 100 µm.

With the change in the expression of cleaved caspase3, Bax and Bcl-2, either IS or cisplatin alone activated apoptosis in LLC and A549 xenograft tumours, whereas combination treatment increased the apoptotic levels compared to using one drug alone (Figure 7A-B).

The proangiogenic factors vascular endothelial factor A (VEGFA) and CD31 were used as endothelial markers to measure angiogenesis in the tumours 26. Similarly, the combination of IS and cisplatin downregulated the expression of VEGFA and CD31 compared with cisplatin treatment (Figure 7C-D). This indicated that the combined therapy was more effective at suppressing angiogenesis in tumour-bearing mice.

### Safety profile of IS in combination with cisplatin *in vivo*

**Figure 8:**
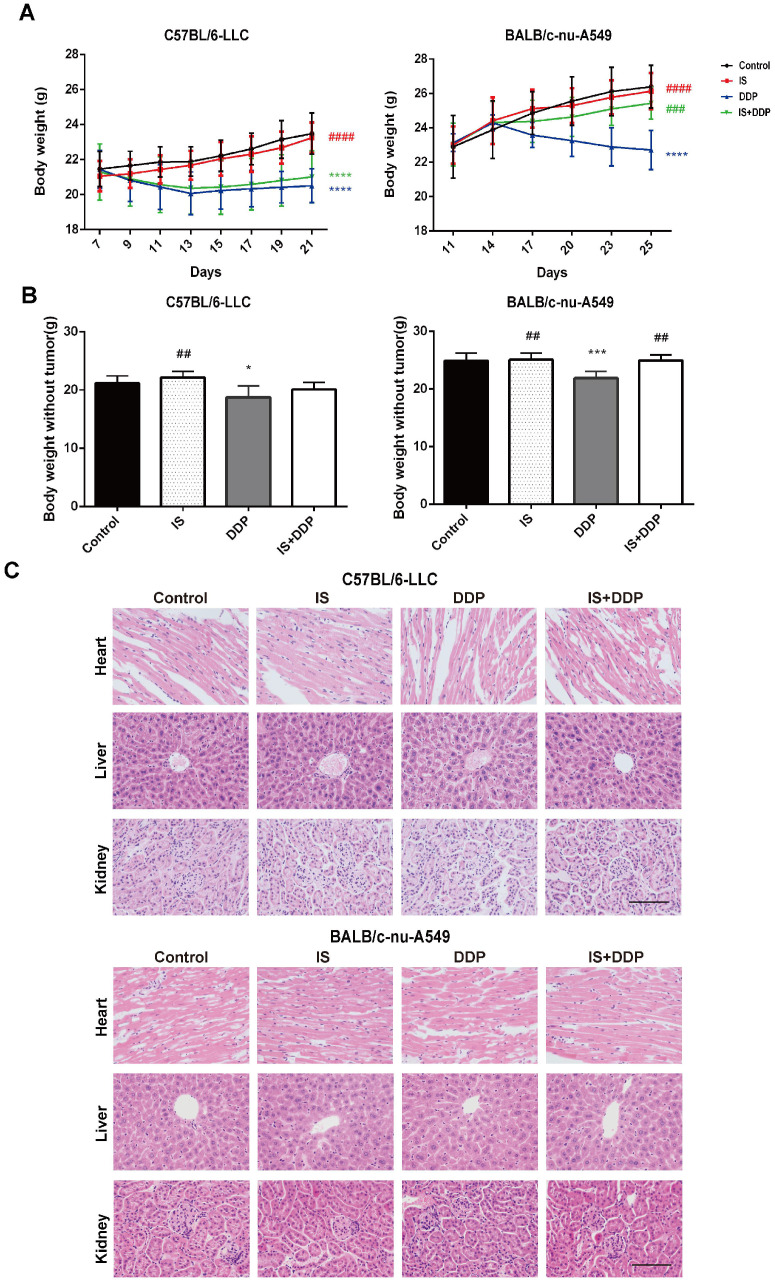
** Icariin II is not toxic to tumour-bearing mice. (A)** Body mass changes of mice in each group during the treatment. **(B)** Body weight of the mice without tumours. **(C)** Representative tissue HE staining of the liver, heart and kidney of the mice after treatment. *P < 0.05, ***P < 0.001, ****P < 0.0001 vs. control group, ##*P* < 0.01, ###*P* < 0.001, ####*P* < 0.0001 vs. the cisplatin group. Scale bar = 100 µm.

We evaluated the biological safety of the combination treatment in mice. As shown in Figure 8A-B, changes in the body weight and net weight without tumours of the mice in different groups were observed. During the treatment process, the body weight of mice treated with IS did not significantly change compared with the control. In the cisplatin-treated groups, a reduction in body weight was observed following injection, which demonstrated the toxic effect of cisplatin. Cotreatment with IS and cisplatin did not affect the body weight when compared with cisplatin treatment in BALB/c mice, and it even increased the body weight in C57BL/6 mice. In addition, histological examination found no evident pathologic changes in the major organs, including the heart, liver and kidney (Figure 8C). No other obvious adverse effects were seen during the combined therapy. These results suggested that the combination strategy caused no toxicity to the mice and might attenuate the adverse effects of cisplatin.

## Discussion

Drug combination therapy has been widely used in cancer to overcome drug resistance or ameliorate drug toxicity 27. The current study demonstrated for the first time the antitumour effect of IS combined with cisplatin on NSCLC. IS enhanced cisplatin cytotoxicity both *in vitro* and *in vivo*. Importantly, our findings suggest that IS combined with cisplatin activated three branches of UPR signalling, thus promoting ER stress-related apoptosis in A549/DDP cells. Our results provide evidence for a new combination strategy of IS and cisplatin for NSCLC treatment.

Our previous study showed the inhibitory effect of IS on LLC tumours, and Song et al. reported the cytotoxicity of IS on A549 cells 28-29. Consistent with these findings, we found that IS suppressed the proliferation of LLC, H1299, A549 and A549/DDP cells in a dose- and time-dependent manner. At 24 h, 48 h and 72 h, the sensitivity of LLC, H1299, and A549 cells to IS were different, while the IC_50_ values of the A549/DDP cells were close to those of the A549 cells. IS had lower cytotoxicity against cells with higher IC_50_ values than cisplatin. The combination treatment of IS and cisplatin significantly inhibited cell proliferation when compared with IS or cisplatin alone. As apoptosis is regarded as the primary mode of cell death induced by most anticancer agents, including cisplatin 27, we next focused on observations of apoptosis progression and the related molecular mechanism.

We used DAPI staining and flow cytometry methods to confirm that IS plus cisplatin induced the most apoptotic cells 48 h posttreatment. Caspase-3 plays a central role in apoptosis, and cleavage of caspase-3 executes the apoptosis program in both the extrinsic and intrinsic pathways in cells 30. Many studies have shown that IS upregulates caspase-3 cleavage in various cancer cells, thus inducing cellular apoptosis and promoting cell death 31-32. The western blot results showed the highest expression of cleaved caspase-3 in the combination treatment group. Taken together, IS promotes the apoptosis induced by cisplatin in all four cell lines.

Furthermore, to explore the mechanism underlying the function of IS, we analysed the gene expression profiles in cisplatin-resistant A549/DDP cells treated with IS with or without cisplatin via mRNA-seq. Consistent with apoptosis upregulation, gene set enrichment analysis demonstrated significant enrichment of apoptosis targets following treatment with the combination. Functional enrichment analysis of the DEGs also showed enrichment related to PERK-mediated UPR signalling. The UPR is the best characterized stress response of the ER and it plays a dual role in cancer. As the ER stress response can trigger the apoptotic signalling pathway, modulation of ER stress is viewed as a potential strategy to treat or restore drug sensitivity in cancers 33. However, whether IS induces ER stress in NSCLC has not yet been studied.

As shown in Figure 4A, IS promoted phosphorylation of PERK and IRE1α and cleavage of ATF6, suggesting activation of all three branches of UPR signalling. In A549/DDP cells receiving combination treatment and 4-PBA, inhibition of ER stress by 4-PBA prevented the expression of the three UPR sensors as well as cleaved caspase-3 while increasing that of Bcl-2. By phosphorylating eIF2α and activating ATF4, PERK induces the expression of CHOP, which is positively connected to apoptosis 20. Both IS and combination treatment triggered PERK-eIF2α-ATF4-CHOP signalling. In summary, we observed the ER stress response induced by IS in NSCLC for the first time, and our results showed that IS might enhance cisplatin-induced apoptosis by promoting ER stress, in which the PERK, IRE1α, ATF6 and PERK-eIF2α-ATF4-CHOP pathways were activated.

Then, we established two NSCLC models to verify the effect of the combination treatment *in vivo*. It was found that the combined therapy significantly reduced the tumour volume and weight in both C57BL/6 and BALB/c mice. Similar to the results in cells, apoptosis assessment of the tumours showed an upregulation of apoptosis in mice treated with IS and cisplatin compared with IS or cisplatin alone. Interestingly, cotreatment facilitated the infiltration of inflammatory cells into LLC tumours and improved tumour angiogenesis in two animal models, which indicated that multiple mechanisms may be involved in the tumour growth inhibition caused by cotreatment. Regarding safety, data on body weight and histological examination of major organs showed promising results. The combination therapy displayed no evident toxicity, and IS may alleviate the side effects of cisplatin.

Platinum-based cytotoxic chemotherapy is currently the preferred first-line option for many advanced NSCLC patients despite recent progress in molecular targeted therapy and immunotherapy. Although cisplatin is one of the most widely used and most potent chemotherapy drugs, as treatment progresses, the likelihood of drug resistance increases. Increasing evidence has revealed the efficacy of natural products in combination with other therapeutic agents. Our results showed that combination therapy of IS and cisplatin is efficacious and safe in NSCLC, which provides new insights into novel therapeutic strategies in cancer treatment.

Several limitations exist in this study. First, IS has been proven to inhibit cancer cell growth by decreasing proliferation, invasion, migration and other mechanisms 34-35. We tested the effects of IS on proliferation and then focused on apoptosis but did not perform experiments to assess invasion or migration, which remains to be studied. Additionally, apoptosis induced by ER stress is an exceedingly complex process induced by combination treatment and it deserves to be explored in more detail. Last, it is necessary to administer these drugs to healthy mice to evaluate further the safety of cotreatment with IS and cisplatin.

In conclusion, we have demonstrated that IS enhances the anticancer efficacy of cisplatin in NSCLC with guaranteed safety. The effect of combined treatment with IS and cisplatin in cells is mainly achieved by upregulation of apoptosis induced by the ER stress response. Considering its efficacy and safety, a combined application of IS and cisplatin might be a promising new strategy for patients with advanced NSCLC.

## Figures and Tables

**Figure 9:**
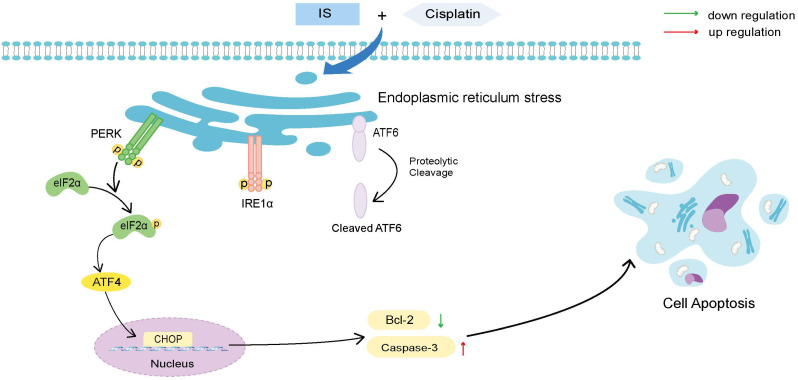
** Schematic illustration of the proposed mechanisms by which IS improves the sensitivity of cancer cells to cisplatin.** IS combined with cisplatin induced ER stress by activating the three branches of UPR signalling and the downstream PERK-eIF2α-ATF4-CHOP pathway, which ultimately enhanced the apoptosis of the cells.

**Table 1:** The IC_50_ values for IS and cisplatin in LLC, H1299, A549 and A549/DDP cells

Drug	Cell line	24 h	48 h	72 h
IS (μmol/L)	LLC	23.82	14.14	12.46
	H1299	37.76	23.18	15.39
	A549	31.97	21.18	16.32
	A549/DDP	32.17	21.98	18.02
DDP (μmol/L)	LLC	16.89	6.68	1.37
	H1299	34.91	22.03	11.68
	A549	22.33	6.71	1.19
	A549/DDP	147.7	48.76	13.28

IS: icariside Ⅱ; DDP: cisplatin. Data were shown with mean ± SD and each experiment was repeated at least three times with minimum three technical replicates.

**Table 2:** The anti-tumor effect of the combined treatment of IS and cisplatin in mice

Mice	Group	Tumor weight (g)	Inhibition rate (%)	*q* value
C57BL/6	Control	1.9 ± 0.21	/	/
	IS	1.51 ± 0.2	20.53	/
	DDP	1.28 ± 0.22	32.63	/
	IS+DDP	0.91 ± 0.16	52.11	1.12
BALB/c-nu	Control	1.49 ± 0.13	/	/
	IS	1.06 ± 0.18	28.86	/
	DDP	0.82 ± 0.23	44.97	/
	IS+DDP	0.49 ± 0.17	67.12	1.1

Data were presented with mean ± SD. n = 5 in each group.

## Abbreviations

4-PBA: 4-phenylbutyric acid
CMC-Na: carboxymethylcellulose sodium
DDP: cisplatin
IS: icariside II
GO: Gene Ontology
DGEs: differentially expressed genes
ER: endoplasmic reticulum
LLC: Lewis lung carcinoma
KEGG: Kyoto Encyclopedia of Genes and Genomes
mRNA-seq: mRNA sequencing
UPR: PERK-mediated unfolded protein response
